# Genetic Determinants for Gestational Diabetes Mellitus and Related Metabolic Traits in Mexican Women

**DOI:** 10.1371/journal.pone.0126408

**Published:** 2015-05-14

**Authors:** Alicia Huerta-Chagoya, Paola Vázquez-Cárdenas, Hortensia Moreno-Macías, Leonardo Tapia-Maruri, Rosario Rodríguez-Guillén, Erika López-Vite, Guadalupe García-Escalante, Fernando Escobedo-Aguirre, Adalberto Parra-Covarrubias, Roberto Cordero-Brieño, Lizette Manzo-Carrillo, Rogelio Zacarías-Castillo, Carlos Vargas-García, Carlos Aguilar-Salinas, Teresa Tusié-Luna

**Affiliations:** 1 Unidad de Biología Molecular y Medicina Genómica, Instituto de Investigaciones Biomédicas, UNAM / Instituto Nacional de Ciencias Médicas y Nutrición Salvador Zubirán, D.F., Mexico City, Mexico; 2 Departamento de Endocrinología y Metabolismo, Instituto Nacional de Ciencias Médicas y Nutrición Salcador Zubirán, D.F., Mexico City, Mexico; 3 Departamento de Economía, Universidad Autónoma Metropolitana, D.F., Mexico City, Mexico; 4 Departamento de Ginelocología y Obstetricia, Hospital General O´Horan, Mérida, Yucatán, México; 5 Departamento de Genética, Universidad Autónoma de Yucatán, Mérida, Yucatán, México; 6 Departamento de Ginecología y Medicina Perinatal, Centro Médico Nacional 20 de Noviembre, D.F., Mexico City, Mexico; 7 Departamento de Endocrinología, Instituto Nacional de Perinatología, D.F., Mexico City, Mexico; 8 Departamento de Ginecología y Obstetricia, Hospital General Manuel Gea González, D.F., Mexico City, Mexico; 9 Departamento de Ginecología y Obstetricia, Centro de Investigación Materno Infantil GEN, D.F., Mexico City, Mexico; Innsbruck Medical University, AUSTRIA

## Abstract

Epidemiological and physiological similarities among Gestational Diabetes Mellitus (GDM) and Type 2 Diabetes (T2D) suggest that both diseases, share a common genetic background. T2D risk variants have been associated to GDM susceptibility. However, the genetic architecture of GDM is not yet completely understood. We analyzed 176 SNPs for 115 *loci* previously associated to T2D, GDM and body mass index (BMI), as well as a set of 118 Ancestry Informative Markers (AIMs), in 750 pregnant Mexican women. Association with GDM was found for two of the most frequently replicated T2D *loci*: a *TCF7L2* haplotype (CTTC: rs7901695, rs4506565, rs7903146, rs12243326; *P*=2.16x10^-06^; OR=2.95) and a *KCNQ1* haplotype (TTT: rs2237892, rs163184, rs2237897; *P*=1.98x10^-05^; OR=0.55). In addition, we found two loci associated to glycemic traits: *CENTD2* (60’ OGTT glycemia: rs1552224, *P*=0.03727) and *MTNR1B* (HOMA B: rs1387153, *P*=0.05358). Remarkably, a major susceptibility *SLC16A11 locus* for T2D in Mexicans was not shown to play a role in GDM risk. The fact that two of the main T2D associated *loci* also contribute to the risk of developing GDM in Mexicans, confirm that both diseases share a common genetic background. However, lack of association with a Native American contribution T2D risk haplotype, *SLC16A11*, suggests that other genetic mechanisms may be in play for GDM.

## Introduction

Gestational Diabetes Mellitus (GDM) is defined as a condition of carbohydrate intolerance of varying severity that begins or is first recognized during pregnancy and is a common obstetric complication. It is characterized by an impaired compensatory increase in insulin secretion to overcome the pregnancy-induced insulin resistance. Nevertheless, the metabolic pathways involved need to be better understood. Previous reports indicate that GDM is a strong risk factor for developing Type 2 Diabetes (T2D) later in life, and most importantly, it also influences the metabolic health of their offspring in the short and long term [1].

In Mexico, no nationwide prevalence has been reported for GDM due to the lack of both detection programs and a consensus diagnostic method. However, a recent study found a prevalence of 12.9% [2], compared to a T2D prevalence of 24.2% among individuals under 40 years old, both reported in 2012 [3]. Physiological similarities between T2D and GDM suggests that they could share a common genetic background. It has been reported that common T2D risk genetic variants confer predisposition to GDM development in Caucasian and Asian populations [4, 5], but up to date, there is scarce information in other ethnic groups. Common T2D risk genetic variants also associated to GDM risk include: *TCF7L2* (rs7903146), *MTNR1B* (rs10830962, rs10830963), *IGF2BP2* (rs4402960, rs1470579), *KCNJ11* (rs5219), *CDKAL1* (rs7754840, rs7756992), *KCNQ1* (rs2237892, rs2237895), *GCK* (rs4607517), *CDKN2A/2B* (rs2383208, rs10811661), *SRR* (rs391300), *HHEX* (rs1111875, rs5015480, rs7923837), *SLC30A8* (rs13266634), *TCF2* (rs7501939).

Although T1D and GDM genetic similarities have been poorly explored, the evidence point out that autoimmunity is not likely to play a major role in GDM development. Although few reports have been published, none of them have shown a high prevalence of T1D risk genotypes in non-autoimmune GDM patients compared to the normoglycemic control group. Moreover, a low percent of GDM patients have shown to be positive to T1D autoimmune markers and pregnancy hyperglycemia are prone to develop T2D, but not T1D [6–9].

It has been shown that ethnicity is an important factor determining the risk of developing metabolic disorders. Particularly, Native American ancestry positively correlates with higher prevalence of T2D and GDM [10–12], a fact that may be related not only to lifestyle, diet and healthcare access, but also to genetic factors. Despite the fact that common T2D susceptibility variants are homogeneous within ancestry groups, their frequency and risk effects have been found to differ across populations [13].

A recent report showed that the inclusion of subjects with high Native American ancestry in a genome wide association study, allowed the identification of new T2D risk alleles, which are rare or absent in other human populations [14]. Furthermore, despite the inclusion of large sample sizes, some of the previously reported T2D associations could not be replicated in Native American derived populations, thus highlighting the importance of assessing the contribution of the currently known risk variants in subjects with different ethnic backgrounds.

In contrast to T2D, genetics of GDM has been less studied. However, a concordance of risk alleles, as well as direction of their effect has been reported. Regarding Mexican population, GDM genetic studies have been conducted for few candidate genes and including small sample sizes. Variants within *TCF7L2*, *TNF* and *HNF4A loci* have been associated to the risk of developing the disease in Mexican women [15–18]. However, no study has evaluated a complete set of the most associated T2D risk variants.

The aim of this work was to analyze whether common genetic variants, previously associated to T2D, obesity and other related traits, were also associated to the risk of GDM in Mexican population.

## Methods

### Ethics Statement

The study was conducted with the approval of the Ethics and Research Committees of the Hospital General Manuel Gea González, Ministry of Health, approval number: 11-86-2010. All participants gave written informed consent before they were included in the study.

### Study participants

Unrelated pregnant Mexican women were recruited at four different health institutions in Mexico City: Instituto Nacional de Perinatología Isidro de los Reyes (INPer), Centro Médico Nacional 20 de Noviembre (CMN20N), Hospital General Manuel Gea González, Centro de Investigación Materno Infantil Gen. INPer is a national referral centre for the treatment of high-risk pregnancy diseases and CMN20M is a white-collar hospital. Both, GEA and CIMIGEN are open-population hospitals, but the second one preferentially provides treatment to pregnant women. All Mexican pregnant (< 20 gestational week) women of 18 years or older were eligible for the study. Exclusion criteria included previous medical diagnosis of T2D or any type of DM, as well as any metabolic disease. After written voluntary consent to participate, it was set an appointment during 24–28 gestational weeks, for a diagnostic OGTT 100 g. Patients were diagnosed following the criteria proposed by Carpenter and Coustan (1982) [19]. A control was defined when no OGTT glucose value was altered, whereas a case was defined when two or more OGTT glucose values were altered. Women who did not finish the diagnostic OGTT or who were diagnosed with a subclinic metabolic disease were excluded. Serum aliquots were immediately frozen after centrifugation for metabolite determinations.

GDM sample consisted in 750 samples (408 cases and 342 controls). Patients were interviewed following a standardized questionnaire, which included demographic, anthropometric, socioeconomic and medical history of the patients and their families. Data from the newborns was also collected.

Given the reported similarities between GDM and T2D, SIGMA T2D Diabetes Consortium data [14] was used for allele effect comparison purposes, preventing association bias given by different sample’s ethnicities. SIGMA T2D Project comprises four study cohorts from Mexico and USA. In order to avoid possible gender and age biases and to ensure an adequate comparison between GDM and SIGMA T2D databases, only women samples were included for both T2D cases and controls. Specifically for cases subgroup, we included women whose age of onset was ≤48 years old, which was the age of the oldest GDM case. The same criteria could not be applied for the control group because SIGMA T2D sample attempted to include hypercontrols, i.e. normoglycemic individuals ≥45 years old. The dataset included 3027 women samples (2467 controls, 560 cases). SIGMA T2D full dataset, which includes 8214 samples (4366 controls, 3848 cases), was also used for final comparisons.

### Biochemical measurements

Glucose, total and HDL cholesterol and triglycerides were measured by enzymatic methods. LDL cholesterol was computed using Friedewald formula. Insulin was determined by radioimmunoassay. Leptin and adiponectin were determined by ELISA.

HOMA index was computed using the HOMA2 calculator published by the University of Oxford, UK (https://www.dtu.ox.ac.uk/homacalculator/). HOMA2 is an updated version of the linear HOMA model, which considers glucose levels ≥24 mM, accounts for renal glucose losses, assumes reduced suppression of hepatic glucose production and increased insulin secretion in response to glucose levels ≥10 mM [20]. Gutt index was computed using the plasma glucose and insulin concentration from fasting and 120 minutes sample from the OGTT, as described by Kanauchi [21].

### SNP selection criteria

In addition to T2D family history, it is well known that a high pregestational BMI is related with an increased risk of developing gestational hyperglycemia and adverse outcomes in the fetus. Thus, we were interested in assessing the relationship between high genetic BMI risk and GDM development. Furthermore, besides conventional glycemic and lipid traits, researchers have analyzed the role of genetic variation on other maternal metabolic phenotypes, as well as neonatal phenotypes. Even when those genetic variants have not been associated to GDM in other populations, we decided to test the possible association of pregnancy related traits to GDM in Mexican women.

In order to achieve this, a literature search was performed, it included independent genetic studies from 2003–2013 identifying and/or replicating *loci* associated with T2D, BMI, GDM and pregnancy related traits [4, 5, 15–18, 22–50]. The dataset was filtered in order to select SNPs that were replicated in at least two populations of different ethnicity and that were either identified through GWAS or had a reported association *P* value<1x10^-04^, if identified through other analysis approach in Mexican or other Latino population. An arbitrary *P* value threshold was established, since most available data in Latinos does not come from GWA studies. The filtered dataset included 195 SNPs, mainly associated to T2D, BMI, dyslipidemia, GDM, and pregnancy traits (105 SNPs, 63 SNPs, 11 SNPs and 16 SNPs, respectively).

Also, a set of 118 AIMs was selected from a published panel of genome wide ancestry informative markers to study admixture throughout the Americas [51]. Prioritization of SNPs included in the set was based in the following criteria: 1–2 AIMs per chromosome, no markers in LD and availability of parental genetic information from public databases. Using genome wide information data available for an independent sample, the 118 AIMs set was validated. Correlation between estimated eigenvectors 1 using either genome wide data or 118 AIMs set was 0.966. Similarly, correlation between Native American ancestry proportions was 0.969 (*P* value <2.2x10^-16^).

### DNA purification and SNP genotyping

Genomic DNA was extracted from whole blood using the QIAmp 96 DNA Blood Kit. Purity and concentration was checked with a NanoDrop ND 100. DNA samples were genotyped at LGC Genomics (Beverly, MA, USA). SNPs with a call rate <97% were considered technical failures at the genotyping facility and were automatically deleted before further quality control.

### Quality control procedure

SNPs with 5% or more missing data within the full dataset or whose call rate between cases and controls was statistically different (*P* value <0.00001) were removed. Subsequently, samples with 10% or more missing data and SNPs with <1% minor allele frequency (MAF) within the full dataset, were also removed. All analyzed SNPs were tested for Hardy Weinberg equilibrium (*P* value <1x10^-5^) using Plink software. SNPs that failed to pass the test were excluded of further analyses. After quality control, dataset included 750 individuals and 294 SNPs from which 118 were AIMs. We analyzed 176 SNPs for 115 *loci*, mainly associated to T2D, BMI, GDM, and pregnancy traits (94 SNPs, 57 SNPs, 10 SNPs and 15 SNPs, respectively) (S5 Table). The final call rate was 0.995.

### Population stratification control

A principal components analysis was performed on the AIMs genotypes, using EIGENSTRAT software [52]. The top 10 principal components were used as covariates for correcting for population stratification, as they accounted for most of the total variance.

STRUCTURE software [53] was used to estimate proportions of Native American ancestry with *K* = 3 clusters. GDM samples were merged with individuals from the Human Genome Diversity Panel (HGDP) dataset including Southern Europeans (Basque, French and Italians), Africans (Mandenka and Yoruba), and Native Americans from Mexico (Pima), but also with Native Americans from the Mexican Genome Diversity Project (MGDP) including Tepehuano, Zapoteca and Maya. The merged dataset included 64 Southern Europeans, 43 Africans and 84 Native Americans.

### Statistical analyses

GDM association analysis was performed via logistic regression using PLINK [54]. Age, pregestational BMI, dummy reference hospital and the top 10 principal components were included as covariates in the regression. In addition, in order to assess the risk allele effect dependent on pregestational BMI, GDM sample was stratified in lean (pregestational BMI<25 kg/m^2^) *vs*. non-lean (pregestational BMI ≥25 kg/m^2^) women. *P* values were corrected using FDR. FDR correction was computed using Plink software, which is based in Benjamini and Hochberg (1995) step-up FDR control.

Quantitative traits association analysis was assessed via linear regression using PLINK. Natural logarithmic transformation was performed when variables distributions were not normal. After running adjusted regression models, residual analyses were conducted to evaluate the adequacy of the models. Age, pregestational BMI, glycemic status, gestational week of diagnosis, dummy reference hospital and the top 10 principal components were included as covariates in the regression. *P* values were corrected using FDR. None of the participants were taking any lipid or glucose medication.

For those *loci* with more than 1 associated SNP, haplotype analysis were performed using PLINK and Haploview [55] software. Linkage disequilibrium was computed and haplotype association analyses were corrected as previously described.

The Quanto v1.2.4 statistical software was used for power calculation [56]. We calculated the power to detect the reported ORs at various risk allele frequencies in additive models, with a sample size of 750 samples (408 cases and 302 controls). The calculations were based on a two-sided alpha of 0.05 and a GDM prevalence of 12%. Statistical power reached at least 90% at risk allele frequencies between 0.2 and 0.8. The risk allele frequencies reported in this study ranged from 0.13 and 0.93.

To compare the allelic frequencies of the associated SNPs among different populations, genetic information from 1000 Genomes Project [57] was used, including Europeans (CEU), Asians (ASN) and Africans (AFR); as well as SIGMA T2D Diabetes Consortium samples [14].

Women-only SIGMA T2D samples, as well as the SIGMA T2D full dataset, were used to make comparisons of the allele size effects using *t* tests. T2D association analysis was also performed via logistic regression using PLINK. Age, BMI and the top 2 principal components were included as covariates in the models.

### SNP Functional Annotation and Gene Set Enrichment Analysis

To identify enriched metabolic pathways among the associated genes, a GSEA was performed using Enrichr [58]. SNPs with a nominal association P value<0.01 were only included. SNP Annotation tool [59] was used in order to obtain functional information of the associated SNPs.

## Results

Our GDM sample included 750 Mexican women (408 cases and 342 controls). Cases were younger than controls and showed a higher BMI (*P*<0.001). After adjusting for age, pregestational BMI and sampling gestational week, cases showed higher triglycerides levels (*P* = 0.001), but lower total HDL and LDL cholesterol levels (*P*<0.001, respectively). Although this finding may be interpreted as a good metabolic control, some authors have hypothesized that it could be due to incapacity to develop the physiological hyperlipidemia of the pregnancy, and that estrogens levels may play an important role [60–62]. Cases were also more insulin resistant, as demonstrated by their higher levels of HOMA IR, glucose, insulin and leptin levels (*P* = 0.002, <0.001, 0.005, 0.007, respectively) and by their lower values of Gutt index and adiponectin levels (*P* = 0.006, 0.002, respectively). Additionally, a higher proportion of cases reported to have family history of diabetes, as well as college education level and employment (*P*<0.001, 0.033, <0.001, respectively) (Table 1). As one of the objectives was to compare our findings to those reported for T2D Mexican individuals, we contrasted our data with that from the SIGMA T2D Consortium study [14] which included 3027 women (560 cases ≤48 years old and 2467 controls).

**Table 1:** GDM dataset description.

	Controls	Cases	*P* value
Sample size	342	408	-
Age (years)	28 [23–34]	35 [31–38]	**<0.001**
Pregestational obesity (%)	10.26	29.68	**<0.001**
Pregestational BMI (kg/m^2^)	23.93 [21.52–26.97]	27.02 [24.4–30.75]	**<0.001**
OGTT gestational age (week)	27.59 [25.22–30.54]	25.41 [21.96–29.02]	**<0.001**
Fasting glucose (mmol/l)	4.16 [4.38–4.72]	4.72 [5.19–5.61]	**<0.001**
Glucose 60' (mmol/l)	6.01 [6.94–7.94]	10.05 [10.66–11.49]	**<0.001**
Glucose 120' (mmol/l)	5.50 [6.11–6.77]	8.92 [9.66–10.43]	**<0.001**
Glucose 180' (mmol/l)	4.88 [5.55–6.19]	6.94 [7.88–8.60]	**<0.001**
Area under the curve (mmol*min/l)	944.01 [1067.27–1166.75]	1505.16 [1585.08–1693.31]	**<0.001**
Fasting insulin (pmol/l)	48 [70.2–96.3]	55.2 [85.8–135.6]	**0.005**
Insulin 120' (pmol/l)	198 [406.8–560.1]	385.8 [512.4–853.2]	0.265
Triglycerides (mmol/l)	2.023 [2.62–3.21]	2.23 [2.89–3.55]	**0.001**
Total cholesterol (mmol/l)	5.47 [6.07–7.0]	4.69 [5.54–6.2]	**0.001**
HDL (mmol/l)	1.50 [1.77–2.10]	1.22 [1.50–1.76]	**<0.001**
LDL (mmol/l)	2.46 [3.06–3.57]	2.03 [2.64–3.27]	**0.001**
Leptin (ng/dl)	21.75 [14.54–30.16]	23.46 [14.01–34.29]	**0.007**
Adiponectin (ng/dl)	10.16 [7.92–12.21]	7.82 [6.55–9.94]	**0.002**
Gutt Index	3.97 [3.33–4.73]	2.67 [2.3–2.98]	**0.006**
HOMA B (%)	170.55 [136.38–206.75]	161 [115.8–213.55]	0.948
HOMA IR	1.5 [1–2]	1.8 [1.1–2.85]	**0.002**
Diabetes family history (%)	68.21	80.15	**<0.001**
Employed (%)	47.12	73.19	**<0.001**
Education ≥college (%)	37.57	50.40	**0.033**
Low socioeconomic status (%)	32.84	44.44	0.272
Native American ancestry	65.4 [56.1–73.8]	63.4 [54.3–74.1]	0.300

It is shown median [25^th^ percentile-75^th^ percentile] or percentages. Controls *vs*. cases comparisons. *P* value of U Mann-Whitney, chi square/Fisher exact test or multiple linear regression for metabolic traits, adjusted for age, pregestational BMI and sampling gestational week.

**Fig 1:**
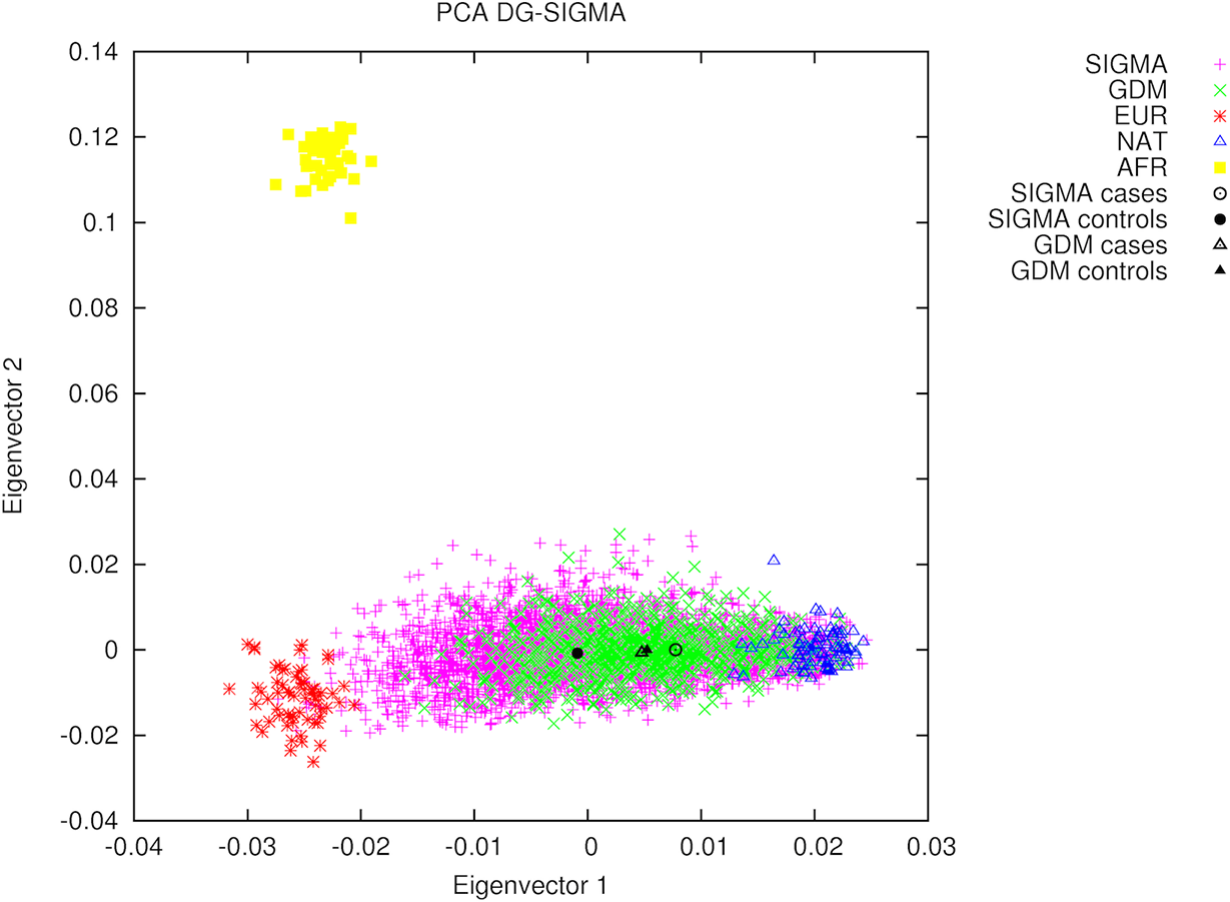
Principal component analysis projection of GDM and SIGMA samples. Principal components were calculated using parental data from the Human Genome Diversity Project (HGDP) and the Mexican Genome Diversity Project (MGDP). Centroids of eigenvectors 1 and 2 for cases and controls are plotted in black triangles for GDM dataset and in black circles for women-only SIGMA dataset.

Even though T2D prevalence has been consistently correlated with a higher proportion of Native American ancestry and low socioeconomic status (SES), no statistical differences between GDM cases and controls were observed (Native American ancestry: 65.4% in controls *vs*. 63.4% in cases, *P* = 0.300; and low SES: 32.8% in controls *vs*. 44.4% in cases, *P* = 0.272) (Table 1). In contrast, women-only SIGMA T2D cases had a higher Native American ancestry proportion when compared to controls (51.8% in controls *vs*. 70.9% in cases, *P* = 1.02x10^-73^) (S1 Fig). Fig 1 shows a PCA projection of GDM and women-only SIGMA T2D samples onto parental populations, as well as the centroids of eigenvectors 1 and 2 for cases and controls. A comparative plot of global ancestry proportions by group is in S1 Fig.

We first examined 176 SNPs previously associated to the risk of T2D, GDM, high BMI and adverse pregnancy traits in other populations. Six variants within two *loci* reached statistical significance for the association to GDM risk after FDR multiple comparisons adjustment (Table 2): *TCF7L2* (rs7901695, *Q* = 0.00314, OR = 2.14[1.47–3.11]; rs4506565, *Q* = 0.00314, OR = 2.06[1.45–2.94]; rs7903146; *Q* = 0.00324, OR = 2.07[1.44–2.98]; rs12243326, *Q* = 0.00061, OR [95%CI] = 2.89[1.85–4.52]) and *KCNQ1* (rs2237892, *Q* = 0.007, OR = 0.58[0.43–0.78]; rs2237897, *Q* = 0.00314, OR = 0.54[0.40–0.72]). The allele effect sizes and frequencies of these SNPs are consistent with previous studies of GDM and T2D [14, 63]. When stratifying by pregestational BMI (lean *vs*. non-lean), association analyses did not reveal a modulation of the *TCF7L2* and *KCNQ1* allele effects sizes (S1 Table). In addition, eight genetic variants showed nominal statistical significance (*P*<0.05): *KLF14* (rs972283), *KCNQ1* (rs163184), *FAIM2* (rs7138803), *HHEX* (rs5015480), *GRB14* (rs3923113), *DUSP9* (rs5945326), *FTSJD1/CALB2* (rs6499500), *PEPD* (rs3786897) (Table 2).

**Table 2:** Genetic variants associated to the risk of GDM in Mexican women.

GENE	SNP	CHR	BP	A1	OR [95% CI]	*P* value	FDR *Q* value
*TCF7L2*	rs7901695	10	114754088	C	2.134 [1.47–3.11]	7.13x10^-05^	**0.00314**
	rs4506565	10	114756041	T	2.06 [1.45–2.94]	6.39x10^-05^	**0.00314**
	rs7903146	10	114758349	T	2.07 [1.44–2.98]	9.20x10^-05^	**0.00324**
	rs12243326	10	114788815	C	2.89 [1.85–4.52]	3.46x10^-06^	**0.00061**
*KCNQ1*	rs2237892	11	2839751	T	0.58 [0.43–0.78]	0.00024	**0.00700**
	rs163184	11	2847069	T	0.70 [0.54–0.91]	0.00774	0.1702
	rs2237897	11	2858546	T	0.54 [0.40–0.72]	4.49x10^-05^	**0.00314**
*KLF14*	rs972283	7	130466854	A	0.66 [0.50–0.87]	0.00303	0.0761
*FAIM2*	rs7138803	12	50247468	A	0.65 [0.47–0.90]	0.01000	0.1956
*HHEX*	rs5015480	10	94465559	C	1.38[1.04–1.84]	0.02619	0.4609
*FTSJD1/CALB2*	rs6499500	16	71348415	C	0.72 [0.54–0.97]	0.03234	0.4635
*GRB14*	rs3923113	2	165501849	G	0.68 [0.48–0.97]	0.03389	0.4635
*DUSP9*	rs5945326	23	152899922	A	1.34 [1.02–1.76]	0.03423	0.4635
*PEPD*	rs3786897	19	33893008	G	1.44 [1.02–2.04]	0.03957	0.4975

Logistic regression corrected for age, pregestational BMI, Native American ancestry and dummy reference hospital.

Next, we tested the haplotypes of the *TCF7L2* and *KCNQ1* for association to GDM and various related metabolic quantitative traits. *TCF7L2* risk haplotype CTTC (*r* ^*2*^ rs7901695 *vs*. rs4506565 = 0.96, *r* ^*2*^ rs7901695 *vs*. rs7903146 = 0.88, *r* ^*2*^ rs7901695 *vs*. rs12243326 = 0.55, *r* ^*2*^ rs4506565 *vs*. rs7903146 = 0.92, *r* ^*2*^ rs4506565 *vs*. rs12243326 = 0.58, *r* ^*2*^ rs7903146 *vs*. rs12243326 = 0.60) was associated to the risk of GDM and to higher levels of fasting glycemia, 60’ and 120’ OGTT glycemia and area under the curve. Similarly, apart from association to GDM, *KCNQ1* protection haplotype TTT (*r* ^*2*^ rs2237892 *vs*. rs163184 = 0.41, *r* ^*2*^ rs2237892 *vs*. rs2237897 = 0.91, *r* ^*2*^ rs163184 *vs*. rs2237897 = 0.39) was also associated to lower levels of 60’ and 180’ OGTT glycemia, and area under the curve (Table 3).

**Table 3:** Haplotypes associated to the risk of GDM and related metabolic traits.

GENE	CHR	LOCATION	KB	SNPs	HAPLOTYPE	*f* controls	*f* cases	TRAIT	OR/B Coeff	*P* value
*TCF7L2*	10	114754088–114788815	34	rs7901695 rs4506565 rs7903146 rs12243326	CTTC	0.0845	0.145	GDM	2.95	**2.16x10** ^**-06**^
								Glucose 0’	0.0308	**0.0128**
								Glucose 60’	0.0971	**2.82x10** ^**-05**^
								Glucose 120’	0.0785	**0.00028**
								Glucose 180’	0.0232	0.342
								AUC	0.0783	**0.000154**
*KCNQ1*	11	2839751–2858546	18.8	rs2237892 rs163184 rs2237897	TTT	0.3406	0.2384	GDM	0.551	**1.98x10** ^**-05**^
								Glucose 0’	-0.0041	0.624
								Glucose 60’	-0.0311	**0.0488**
								Glucose 120’	-0.0195	0.184
								Glucose 180’	-0.0474	**0.00381**
								AUC	-0.0312	**0.0263**

Logistic regression corrected for age, pregestational BMI, Native American ancestry and dummy reference hospital; or multiple linear regression corrected for age, pregestational BMI, OGTT gestational week, Native American ancestry and dummy reference hospital. OR or B coefficient is shown, respectively. AUC is the OGTT Area Under the Curve.

We then examined the 176 SNPs for association to fluctuations of glycemic metabolic quantitative traits. Two variants reached statistical significance after FDR multiple comparisons adjustment: *CENTD2* (60’ OGTT glycemia: rs1552224, *Q* = 0.02599), *MTNR1B* (HOMA B: rs1387153, *Q* = 0.03881) (Table 4 and S2 Table). Interestingly, *MTNR1B* rs1387153 variant was nominally associated to GDM in non-lean women subset (lean: OR = 0.888, *P* = 0.6337 *vs*. non-lean: OR = 1.761, P = 0.01297, t test *P* value = 0.0432) (S1 Table).

**Table 4:** Genetic variants significantly associated with metabolic trait fluctuations in pregnant Mexican women.

GENE	SNP	CHR	BP	A1	TRAIT	B Coeff	*P* value	FDR *Q* value
*CENTD2*	rs1552224	11	72433098	T	Glucose 60'	0.1118	0.00061	**0.02699**
*MTNR1B*	rs1387153	11	92673828	T	HOMA B	-0.1176	0.00022	**0.03881**

Multiple linear regressions corrected for age, pregestational BMI, glycemic status, OGTT gestational week, Native American ancestry and dummy reference hospital.

We were also interested in comparing the risk effects of *TCF7L2* and *KCNQ1* variants between GDM and T2D. For this purpose, we compared the Beta coefficients obtained in the T2D case-control group from SIGMA T2D Consortium study [14] with those obtained in GDM. As previously reported, both *loci* reached genome-wide significant association to T2D in Mexican population, *TCF7L2* (rs7903146, *P* = 2.5x10^-17^, OR [95%CI] = 1.41[1.30–1.53]) and *KCNQ1* (rs2237897, *P* = 4.9x10^-16^, OR = 0.74[0.69–0.80]) [14]. The effect of *TCF7L2* risk alleles was significantly different between the women-only T2D and GDM patients (t test *P*<0.01). In contrast, no difference was found in the risk effect of the *KCNQ1* variants between the two groups (t test *P*>0.1) (Table 5). S3 Table shows allelic frequencies for the analyzed datasets.

**Table 5:** Risk allele effect comparison between GDM and T2D samples.

			GDM (N = 408/342)		Women-only SIGMA T2D ≤48 years (N = 2467/560)			SIGMA T2D (N = 4366/3848)	
**GENE**	**SNP**	**A1**	**OR**	***P* value**	**OR**	***P* value**	**t test *P* value**	**OR**	***P* value**
*TCF7L2*	rs7901695	C	2.14	**7.13x10** ^**-05**^	1.17	0.1064	**0.0048**	1.33	**4.76x10** ^**-13**^
	rs4506565	T	2.06	**6.39x10** ^**-05**^	1.16	0.1186	**0.0075**	1.35	**2.51x10** ^**-14**^
	rs7903146	T	2.07	**9.20x10** ^**-05**^	1.21	0.05725	**0.0136**	1.41	**2.50x10** ^**-17**^
	rs12243326	C	2.89	**3.46x10** ^**-06**^	1.17	0.1566	**0.0003**	1.35	**1.02x10** ^**-11**^
*KCNQ1*	rs2237892	T	0.58	**2.39x10** ^**-04**^	0.73	**1.74x10** ^**-04**^	0.2113	0.78	**1.08x10** ^**-12**^
	rs2237897	T	0.54	**4.49x10** ^**-05**^	0.68	**7.26x10** ^**-06**^	0.2052	0.74	**4.9x10** ^**-16**^

Logistic regression corrected for age, BMI and Native American ancestry. **t test *P* value** of comparison between risk allele effect of GDM and women-only SIGMA T2D ≤48 years samples. **N** is the sample size of controls/cases used in the analyses.

Despite the association with GDM observed for *TCF7L2* and *KCNQ1*, no association was found for *SLC16A11* haplotype, which was recently identified as a major contributor for T2D risk in Mexican population [14]. Even though the frequency of *SLC16A11* risk variants in GDM cases was found to be similar to that of women-only SIGMA T2D cases (*P*>0.1), no statistical difference was found for the *SLC16A11* risk variants between GDM cases and controls (*P*>0.1) (S3 Table). As all association analyses included a previously validated set of AIMs, lack of association was not due to a deficient population stratification control.

Finally, considering that variants near or within 59 gene *loci* showed nominal association to the risk of GDM or to metabolic and pregnancy related traits, we performed a Gene Set Enrichment Analysis (GSEA) using an association *P* value threshold <0.01. Besides evident related GO biological processes (i.e. glucose homeostasis, negative regulation of signal transduction, positive regulation of metabolic process, regulation of insulin secretion, generation of precursor metabolites, energy reserve metabolic process, feeding behavior), the analysis revealed two additional interesting processes: transcription regulation from RNA polymerase II promoter and gene-specific transcription (*P*<0.05). It is noteworthy that all genetic variants that reached statistical significance after FDR multiple comparisons adjustment, are located either in coding untranslated regions or within non-coding sites (S4 Table).

## Discussion

Our results support a common genetic background for T2D and GDM. Not only did we find *TCF7L2* and *KCNQ1* variants to be associated to GDM, but GDM cases reported higher frequency of family history of diabetes than controls. In the recent SIGMA T2D GWAS in Mexicans, it was demonstrated that common variants within the *TCF7L2* and *KCNQ1* genes were among the strongest associated *loci* [14]. In the current study, associated genetic variants in these two *loci* were directionally consistent with T2D results previously reported for Mexicans and other populations [5, 14, 15, 64]. Also, we found that allele effect sizes of *TCF7L2* variants were significantly different between GDM and T2D, suggesting a major risk effect of this *locus* in the development of GDM. Additionally, when analyzed variants within *TCF7L2* and *KCNQ1* were grouped in LD blocks, the *TCF7L2* risk haplotype was significantly associated with increased glucose levels during OGTT; whereas, the *KCNQ1* protection haplotype, was associated with decreased glycemia.

*TCF7L2* gene encodes for the TCF4 transcription factor, involved in the Wnt signaling pathway. Risk variants are located in an intronic 92 kb interval, which has been reported to confer the strongest effect on T2D risk among the more than 90 T2D associated *loci* to date. Despite the mechanisms through which *TCF7L2* influences disease development have not been fully understood, it has been proposed to be related to a reduced insulin secretion. One study showed that the rs7903146 risk allele T is located within a region of open chromatin and increased enhancer activity [65], thus increasing gene expression in pancreatic islets and presumably other tissues [66]. Given that others have reported downregulation of *TCF7L2* gene expression, risk variants have been proposed to alter *TCF7L2* splicing patterns, suggesting that functionally distinct mRNA isoforms, rather than levels of expression, define their phenotypic consequences [67]. In the case of GDM, expression of *TCF7L2* was decreased in visceral adipose tissue from women who developed hyperglycemia, although after adjusting for BMI, the difference was not significant [68].

*KCNQ1* gene encodes the Kv7.1 voltage-gated potassium channel. Even though it is known to play an important role in shaping the cardiac action potential, as well as in controlling the water and salt homeostasis in epithelial tissues [69], its role in diabetes development is not yet clear. *KCNQ1* is located at the imprinted 11p15.4 region and its expression is monoallelic during early embryogenesis but turns biallelic during fetal heart development [70]. Two independent signals within introns 11 and 15 have been associated to T2D development, but only when risk alleles are maternally inherited [71]. *KCNQ1* region is regulated by differential methylation at the promoter of *KCNQ1OT1*, a paternally expressed non-coding antisense RNA, which results in maternal expression of neighbouring genes. Risk allele of rs2237895 located within intron 15 was associated with impaired exocytosis and a reduced amount of docked granules of insulin, especially among non-obese allele carriers [72]. The same risk allele was reported to be associated with hypermethylation of the DMR and CTCF binding region near *KCNQ1OT1* in fetal pancreas, but no relationship with risk allele number and gene expression was found [71].

Apart from *TCF7L2* and *KCNQ1*, seven additional *loci* previously related to T2D (*KLF14*, *HHEX*, *GRB14*, *DUSP9* and *PEPD*), increased BMI (*FAIM2*) or GDM (*FTSJD1/CALB2*), were nominally associated to the risk of developing GDM. In contrast to T2D genetic studies, those performed for GDM have yielded scarce results. This is due in part, to the inclusion of smaller sample sizes, sample heterogeneity as a result of different inclusion criteria and diagnostic methods. Although most of GDM genetic studies have been preferentially conducted for candidate genes, meta-analyses have consistently revealed variants near previously T2D associated genes, i.e. *TCF7L2*, *MTNR1B*, *IGF2BP2*, *KCNJ11*, *CDKAL1*, *KCNQ1*, *GCK* and *IRS1* [63, 64]. In the only published GWAS for GDM performed in Korean women, the authors found association to common T2D variants and identified novel signals with marginal association [5]. It is of interest that one of them, *FTSJD1/CALB2*, was also nominally associated in our sample.

Of particular relevance is the finding of a differential role of *SLC16A11* gene *locus* in the susceptibility to T2D and GDM. It was recently reported that *SLC16A11* has a major contribution to T2D risk in Native American derived populations, as risk associated variants are found at a substantially higher frequency in individuals with Native American ancestry [14]. In the present study, no statistical difference was observed in Native American ancestry proportions between GDM cases and controls and consequently similar allele frequencies for *SLC16A11* risk associated variants between both groups were observed. This finding suggest that apart from T2D common gene variants, other genetic and possibly environmental risk factors may play a role in the development of GDM. The association of *FTSJD1/CALB2 locus* with GDM risk in two independent studies supports the fact that other GDM susceptibility *loci* are yet to be identified.

Low SES has also been associated to the risk of developing T2D [12], yet similar proportions of GDM cases and controls were classified in the low SES category. It is also noteworthy that a major proportion of cases reported to be college graduates and to be employed. These findings suggest that environmental risk factors, rather than Native American ancestry are important determinants of GDM susceptibility. Reported environmental factors associated with the risk of developing GDM include excess high energy and saturated fat intakes [73], as well as dietary deficiencies of vitamins B12 and 25OHD [74, 75].

Besides the relationship between the common T2D gene risk variants with GDM, we also found association of two other T2D related *loci* with increased 60’ OGTT glucose levels (*CENTD2*) and decreased HOMA B (*MTNR1B*). The *CENTD2* LD region comprises at least three other genes (*PDE2A*, *QTG16L2*, *FCHSD2*). Because none of them have been implicated in insulin processing or secretion, *CENTD2*, also known as *ARAP1*, is proposed to be the responsible gene within this *locus*. It encodes a protein which activates Arf and Rho GTPases to regulate membrane trafficking and actin cytoskeleton reorganization. Apart from T2D, this risk *locus* has been strongly associated to fasting proinsulin and 32-33-split proinsulin. Recently, it was proposed that rs11603334, which is in perfect LD with rs1552224 (analyzed in this study), is a functional variant regulating *ARAP1* expression. The variant rs11603334 is located at the P1 promoter and overlaps transcription factor binding sites within a region of open chromatin that is marked by DNA hypersensitivity, H3K4me3 and H3K9ac in pancreatic islets. Risk allele C disrupts binding of PAX4/6 transcriptional factors and increases transcriptional activity by 2-fold, thus leading to higher gene expression [76]. PAX4/6 are known regulators of both glucagon and insulin promoters. Coding variants within them have been previously implicated in glucose homeostasis [77].

As to the role of *MTNR1B*, genetic variants within this *locus* have been associated to an increase in fasting glucose levels, reduced beta cell function, as well as T2D and GDM risk [35]. Circadian rhythms have been recognized as critical for glucose homeostasis maintenance and melatonin is among their major regulators. Melatonin is a diurnal hormone produced by the pineal gland, which binds to melatonin receptor 2, encoded by *MTNR1B* gene, to exert its functions. Even though the causal variant is yet to be identified, intronic variant rs10830963 is the strongest associated signal. No evidence has been reported regarding its molecular mechanism, but it does not seem to disrupt consensus transcription factor binding or cryptic alternative splice sites. Nevertheless, risk allele G was associated to *MTNR1B* increased gene expression in pancreatic islets [78], thus impairing insulin secretion. Of particular importance is the fact that during pregnancy, plasma melatonin levels are elevated and it crosses the placenta and fetal blood-brain barrier, playing a key role not only in the prevention of pregnancy loss, but also in the development of fetal organs and its adaptation to extra-uterine life [79].

GWAS have allowed an accelerated identification of T2D risk allele variants in a short period of time. Nevertheless, we are still missing the mechanisms by which they confer risk to the disease. It is remarkable that all associated variants are located within non-coding regions and their nearest corresponding gene belong to GO terms involved in the regulation of gene transcription and further transcript abundance, with a higher frequency that may occur by chance. Two interesting enriched GO terms were regulation of transcription from RNA polymerase II promoter and gene-specific transcription, which emphasize the putative regulatory function of involved genes. More studies are needed to clarify the mechanisms involved on disease susceptibility. Apart from T2D, our results pointed at other implicated metabolic pathways and transcriptional regulatory mechanisms, thus highlighting the complex pathophysiology of GDM. Genome-wide studies with larger sample size are still needed to identify genes with smaller allele effects and possibly GDM specific risk variants. Additional analyses, such as global expression studies in tissues relevant to disease physiology, will also be required to elucidate the role of different gene risk variants in disease susceptibility.

## Supporting Information

S1 Fig Global ancestry proportions plot of GDM and SIGMA samples, as well as parental samples from The Human Genome Diversity Project (HGDP) and The Mexican Genome Diversity Project (MGDP). (DOCX)

S1 Table Risk allele effect comparison between lean and non-lean GDM samples. (DOCX)

S2 Table Genetic variants associated to metabolic trait fluctuations during pregnancy in Mexican women. (DOCX)

S3 Table Allele frequencies of the genetic variants associated with the risk of GDM and related metabolic traits in Mexican women, as well as *SLC16A11 loci*. (DOCX)

S4 Table Functional annotation of the genetic variants associated to the risk of GDM and related metabolic traits in Mexican women. (DOCX)

S5 Table SNPs analyzed in current study. (DOCX)
