# Supplementary material for: Genetic Determinants for Gestational Diabetes Mellitus and Related Metabolic Traits in Mexican Women
Source: PLoS One. 2015 May 14;10(5):e0126408. doi: 10.1371/journal.pone.0126408 (PMC4431878; doi:10.1371/journal.pone.0126408)
Supplement: S1 Fig — (DOCX) [file pone.0126408.s001.docx]

**S1 Figure. Global ancestry proportions plot of GDM and SIGMA samples, as well as parental samples from The Human Genome Diversity Project (HGDP) and The Mexican Genome Diversity Project (MGDP).**


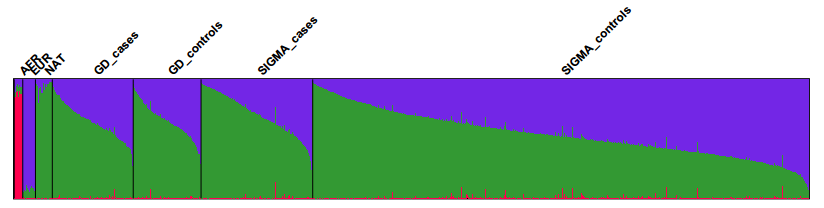


| **Native American ancestry proportion (%)** | | | |
| --- | --- | --- | --- |
|  | **Controls** | **Cases** | ***P* value** |
| **GDM** | 65.42 [56.12-73.82] | 63.38 [54.29-74.08] | 0.3004 |
| **SIGMA T2D** | 51.83 [39.67-66.35] | 70.86 [57.58-84.86] | 1.02x10^-73^ |
| *It is shown median [25^th^ percentile-75^th^ percentile]. | | | |
