# Supplementary material for: Genetic Determinants for Gestational Diabetes Mellitus and Related Metabolic Traits in Mexican Women
Source: PLoS One. 2015 May 14;10(5):e0126408. doi: 10.1371/journal.pone.0126408 (PMC4431878; doi:10.1371/journal.pone.0126408)
Supplement: S1 Table — (DOCX) [file pone.0126408.s002.docx]

| **S1 Table. Risk allele effect comparison between lean and non-lean GDM samples.** | | | | | | | |  |
| --- | --- | --- | --- | --- | --- | --- | --- | --- |
|  |  |  | **Lean**  (N=184/125) | | **Non-Lean**  (N=128/248) | |  | |
| **GENE** | **SNP** | **A1** | **OR** | ***P* value** | **OR** | ***P* value** | **t test *P* value** | |
| *TCF7L2* | rs7901695 | C | 2.649 | **0.001768** | 1.897 | **0.009695** | 0.3957 | |
|  | rs4506565 | T | 2.211 | **0.005541** | 2.03 | **0.003126** | 0.8176 | |
|  | rs7903146 | T | 2.365 | **0.003477** | 1.956 | **0.00634** | 0.618 | |
|  | rs12243326 | C | 2.473 | **0.01136** | 3.327 | **9.87x10^-05^** | 0.5284 | |
| *KCNQ1* | rs2237892 | T | 0.6298 | **0.04575** | 0.5216 | **0.0008519** | 0.5307 | |
|  | rs2237897 | T | 0.6123 | **0.03994** | 0.4698 | **0.0002011** | 0.3956 | |
| *CENTD2* | rs1552224 | T | 1.154 | 0.7406 | 1.573 | 0.2567 | 0.5999 | |
| *MNTR1B* | rs1387153 | T | 0.888 | 0.6337 | 1.761 | **0.01297** | **0.0432** | |
| Logistic regression corrected for age, Native American ancestry and dummy reference hospital. **t test *P* value** of comparison between risk allele effect of lean and non-lean GDM samples. **N** is the sample size of controls/cases used in the analyses. | | | | | | | |  |
