# Supplementary material for: Genetic Determinants for Gestational Diabetes Mellitus and Related Metabolic Traits in Mexican Women
Source: PLoS One. 2015 May 14;10(5):e0126408. doi: 10.1371/journal.pone.0126408 (PMC4431878; doi:10.1371/journal.pone.0126408)
Supplement: S2 Table — (DOCX) [file pone.0126408.s003.docx]

| **S2 Table. Genetic variants associated to metabolic trait fluctuations during pregnancy in Mexican women.** | | | | | | | | |
| --- | --- | --- | --- | --- | --- | --- | --- | --- |
| **GENE** | **SNP** | **CHR** | **BP** | **A1** | **TRAIT** | **B COEFF.** | ***P* value** | **FDR *Q* value** |
| *TCF7L2* | rs7901695 | 10 | 114754088 | C | Glucose 0' | 0.028 | 0.00645 | 0.316 |
|  |  |  |  |  | Glucose 60' | 0.070 | 0.00035 | **0.02052** |
|  |  |  |  |  | Glucose 120' | 0.069 | 0.00016 | **0.02208** |
|  |  |  |  |  | Glucose 180' | 0.014 | 0.4809 | 0.8872 |
|  |  |  |  |  | AUC | 0.063 | 0.00032 | **0.02783** |
|  | rs4506565 | 10 | 114756041 | T | Glucose 0' | 0.030 | 0.00223 | 0.316 |
|  |  |  |  |  | Glucose 60' | 0.066 | 0.00030 | **0.02052** |
|  |  |  |  |  | Glucose 120' | 0.063 | 0.00026 | **0.02208** |
|  |  |  |  |  | Glucose 180' | 0.014 | 0.4572 | 0.8872 |
|  |  |  |  |  | AUC | 0.056 | 0.00066 | **0.03897** |
|  | rs7903146 | 10 | 114758349 | T | Glucose 0' | 0.027 | 0.00804 | 0.316 |
|  |  |  |  |  | Glucose 60' | 0.056 | 0.00287 | 0.1009 |
|  |  |  |  |  | Glucose 120' | 0.057 | 0.00118 | 0.05209 |
|  |  |  |  |  | Glucose 180' | 0.015 | 0.4355 | 0.8872 |
|  |  |  |  |  | AUC | 0.048 | 0.00438 | 0.1542 |
|  | rs12243326 | 10 | 114788815 | C | Glucose 0' | 0.026 | 0.02385 | 0.4546 |
|  |  |  |  |  | Glucose 60' | 0.093 | 2.09x10^-05^ | **0.00367** |
|  |  |  |  |  | Glucose 120' | 0.072 | 0.00038 | **0.02208** |
|  |  |  |  |  | Glucose 180' | 0.030 | 0.1951 | 0.8499 |
|  |  |  |  |  | AUC | 0.082 | 2.35x10^-05^ | **0.004126** |
| *KCNQ1* | rs2237892 | 11 | 2839751 | T | Glucose 0' | -0.004366 | 0.5975 | 0.9933 |
|  |  |  |  |  | Glucose 60' | -0.02126 | 0.1725 | 0.9787 |
|  |  |  |  |  | Glucose 120' | -0.01386 | 0.338 | 0.8837 |
|  |  |  |  |  | Glucose 180' | -0.04044 | 0.01254 | 0.2758 |
|  |  |  |  |  | AUC | -0.02594 | 0.06132 | 0.5396 |
|  | rs163184 | 11 | 2847069 | T | Glucose 0' | -0.01329 | 0.08464 | 0.8674 |
|  |  |  |  |  | Glucose 60' | -0.02537 | 0.08102 | 0.7083 |
|  |  |  |  |  | Glucose 120' | -0.01143 | 0.3979 | 0.8862 |
|  |  |  |  |  | Glucose 180' | -0.03967 | 0.007793 | 0.2743 |
|  |  |  |  |  | AUC | -0.02455 | 0.05802 | 0.5375 |
|  | rs2237897 | 11 | 2858546 | T | Glucose 0' | -0.005271 | 0.5317 | 0.9933 |
|  |  |  |  |  | Glucose 60' | -0.03025 | 0.05614 | 0.6845 |
|  |  |  |  |  | Glucose 120' | -0.01925 | 0.1918 | 0.8837 |
|  |  |  |  |  | Glucose 180' | -0.04721 | 0.004042 | 0.2315 |
|  |  |  |  |  | AUC | -0.03186 | 0.02388 | 0.3821 |
| *CENTD2* | rs1552224 | 11 | 72433098 | T | Glucose 0' | 0.008 | 0.6552 | 0.316 |
|  |  |  |  |  | Glucose 60' | 0.112 | 0.00061 | **0.02699** |
|  |  |  |  |  | Glucose 120' | 0.029 | 0.3411 | 0.8837 |
|  |  |  |  |  | Glucose 180' | 0.038 | 0.2618 | 0.8499 |
|  |  |  |  |  | AUC | 0.072 | 0.01298 | 0.3368 |
| *MTNR1B* | rs1387153 | 11 | 92673828 | T | HOMA B | -0.11760 | 0.00022 | **0.03881** |
|  |  |  |  |  | HOMA IR | -0.08654 | 0.06071 | 0.834 |
|  |  |  |  |  | HOMA S | 0.08714 | 0.05827 | 0.8151 |
|  |  |  |  |  | Insulin | -0.08673 | 0.06029 | 0.8843 |
|  | rs10830963 | 11 | 92708710 | G | HOMA B | -0.09819 | 0.00208 | 0.09167 |
|  |  |  |  |  | HOMA IR | -0.08687 | 0.06105 | 0.834 |
|  |  |  |  |  | HOMA S | 0.08758 | 0.05834 | 0.8151 |
|  |  |  |  |  | Insulin | -0.07896 | 0.08453 | 0.9298 |
| Multiple linear regression corrected for age, pregestational BMI, glycemic status, OGTT gestational week, Native American ancestry and dummy reference hospital. | | | | | | | | |
