# Supplementary material for: Genetic Determinants for Gestational Diabetes Mellitus and Related Metabolic Traits in Mexican Women
Source: PLoS One. 2015 May 14;10(5):e0126408. doi: 10.1371/journal.pone.0126408 (PMC4431878; doi:10.1371/journal.pone.0126408)
Supplement: S3 Table — (DOCX) [file pone.0126408.s004.docx]

| **S3 Table. Allele frequencies of the genetic variants associated with the risk of GDM and related metabolic traits in Mexican women, as well as *SLC16A11 loci*.** | | | | | | | | | | | |
| --- | --- | --- | --- | --- | --- | --- | --- | --- | --- | --- | --- |
|  |  |  | **GDM**  (N=342/408) | | | **SIGMA T2D**  (N=4366/3848) | | | **1 KG** | | |
|  |  |  |  |  |  |  |  |  |  |  |  |
| **GENE** | **SNP** | **A1** | **Cases** | **Controls** | ***P* value** | **Cases** | **Controls** | ***P* value** | **EUR** | **AFR** | **ASN** |
| *TCF7L2* | rs7901695 | C | 0.203 | 0.136 | **1.5x10^-03^** | 0.245 | 0.216 | **0.0001** | 0.325 | 0.460 | 0.044 |
|  | rs7903146 | T | 0.199 | 0.127 | **9.0x10^-04^** | 0.232 | 0.196 | **2.5x10^-07^** | 0.320 | 0.280 | 0.039 |
|  | rs4506565 | T | 0.213 | 0.140 | **1.3x10^-03^** | 0.244 | 0.214 | **3.8x10^-05^** | 0.332 | 0.460 | 0.044 |
|  | rs12243326 | C | 0.168 | 0.096 | **1.0x10^-04^** | 0.191 | 0.171 | **0.0056** | 0.279 | 0.270 | 0.023 |
| *KCNQ1* | rs2237897 | T | 0.247 | 0.344 | **2.0x10^-04^** | 0.246 | 0.277 | **5.2x10^-05^** | 0.042 | 0.080 | 0.366 |
|  | rs2237892 | T | 0.261 | 0.349 | **0.0012** | 0.263 | 0.287 | **0.0025** | 0.060 | 0.110 | 0.333 |
| MTNR1B | rs1387153 | T | 0.233 | 0.204 | 0.238 | 0.222 | 0.222 | 0.7974 | 0.269 | 0.400 | 0.438 |
| *CENTD2* | rs1552224 | T | 0.954 | 0.939 | 0.4328 | 0.942 | 0.929 | **0.0055** | 0.866 | 0.990 | 0.930 |
| *SLC16A11* | rs13342232 | G | 0.367 | 0.365 | 0.4866 | 0.351 | 0.283 | **6.9x10^-19^** | 0.039 | 0.010 | 0.120 |
|  | rs13342692 | C | 0.365 | 0.367 | 0.5345 | 0.350 | 0.283 | **1.3x10^-18^** | 0.039 | 0.010 | 0.120 |
|  | rs117767867 | T | 0.353 | 0.355 | 0.6461 | 0.328 | 0.263 | **6.2x10^-23^** | 0.028 | 0.010 | 0.120 |
|  | rs75418188 | T | 0.354 | 0.359 | 0.5565 | 0.330 | 0.264 | **4.4x10^-18^** | 0.032 | 0.010 | 0.120 |
|  | rs75493593 | T | 0.356 | 0.357 | 0.7619 | 0.330 | 0.264 | **3.1x10^-18^** | 0.028 | 0.010 | 0.120 |
| **A1**, frequency reported allele. ***P* value** of the chi exact or Fisher test cases *vs.* controls allele frequencies comparison. **N** is the sample size of controls/cases used in the analyses. **EUR** (European), **AFR** (African) and **ASN** (Asian) allele frequencies are from publicly available results from the **1KG** Project (1000 Genomes Project). | | | | | | | | | | | |
