# Supplementary material for: Genetic Determinants for Gestational Diabetes Mellitus and Related Metabolic Traits in Mexican Women
Source: PLoS One. 2015 May 14;10(5):e0126408. doi: 10.1371/journal.pone.0126408 (PMC4431878; doi:10.1371/journal.pone.0126408)
Supplement: S4 Table — (DOCX) [file pone.0126408.s005.docx]

| **S4 Table. Functional annotation of the genetic variants associated to the risk of GDM and related metabolic traits in Mexican women.** | | | | | | | | |
| --- | --- | --- | --- | --- | --- | --- | --- | --- |
| **GENE** | **CHR** | **BAND** | **SNP** | **ALLELE** | **STRAND** | **PREDICTED FUNCTION** | **cDNA POS** | **SPLICE DISTANCE** |
| *CENTD2* | 11 | q13.4 | rs1552224 | A\|C | 1 | 5’UTR | 306 | NA |
| *KCNQ1* | 11 | p15.4 | rs2237892 | C\|T | 1 | Intronic | NA | 29246 |
|  |  |  | rs2237897 | C\|T | 1 | Intronic | NA | 10451 |
| *MTNR1B* | 11 | q14.3 | rs1387153 | C\|T | 1 | Intergenic | NA | NA |
| *TCF7L2* | 10 | q25.2 | rs12243326 | T\|C | 1 | Intronic | NA | 10969 |
|  |  |  | rs4506565 | A\|T | 1 | Intronic | NA | 31658 |
|  |  |  | rs7901695 | T\|GCA | 1 | Intronic | NA | 29705 |
|  |  |  | rs7903146 | C\|T | 1 | Intronic | NA | 33966 |
| **ALLELE:** Examined alleles (reference allele/observed allele)  **STRAND:** 1, forward; -1, reverse  **PREDICTED FUNCTION:** Predicted function of the SNP based on its location on the transcript  **cDNA POS:** SNP position on cDNA, if the prediction function is coding, 3’ UTR or 5’ UTR  **SPLICE DISTANCE:** Distance to splice junction, if the predicted function is intronic  * rs7901695 studied alleles are T/C | | | | | | | | |
