# Supplementary material for: Genetic Determinants for Gestational Diabetes Mellitus and Related Metabolic Traits in Mexican Women
Source: PLoS One. 2015 May 14;10(5):e0126408. doi: 10.1371/journal.pone.0126408 (PMC4431878; doi:10.1371/journal.pone.0126408)
Supplement: S5 Table — (DOCX) [file pone.0126408.s006.docx]

| **S5 Table. SNPs analyzed in current study.** | | | | | | |
| --- | --- | --- | --- | --- | --- | --- |
| **SNP** | **CHR** | **BP** | **ALLELES** | **GENE** | **TRAIT** | **REFERENCE** |
| rs1501299 | 3 | 186571123 | A/C | *ADIPOQ* | BMI | [36] |
| rs266729 | 3 | 186559474 | C/G | *ADIPOQ* | BMI | [36] |
| rs2030323 | 11 | 27728539 | G/T | *BDNF* | BMI | [45] |
| rs10767664 | 11 | 27725986 | A/T | *BDNF* | BMI | [48] |
| rs4923461 | 11 | 27656910 | A/G | *BDNF* | BMI | [49] |
| rs6265 | 11 | 27679916 | A/G | *BDNF* | BMI | [56] |
| rs13078807 | 3 | 85884150 | A/G | *CADM2* | BMI | [48] |
| rs9816226 | 3 | 185834499 | A/T | *ETV5* | BMI | [48] |
| rs7647305 | 3 | 185834290 | C/T | *ETV5* | BMI | [49] |
| rs7138803 | 12 | 50247468 | A/G | *FAIM2* | BMI | [48] |
| rs887912 | 2 | 59302877 | A/G | *FANCL* | BMI | [48] |
| rs2112347 | 5 | 75015242 | G/T | *FLJ35779* | BMI | [48] |
| rs9939609 | 16 | 53820527 | A/T | *FTO* | BMI | [38] |
| rs1121980 | 16 | 53809247 | C/T | *FTO* | BMI | [43] |
| rs12149832 | 16 | 53842908 | A/G | *FTO* | BMI | [45] |
| rs11642841 | 16 | 53845487 | A/C | *FTO* | BMI | [46] |
| rs1421085 | 16 | 53800954 | C/T | *FTO* | BMI | [8] |
| rs9941349 | 16 | 53825488 | C/T | *FTO* | BMI | [8] |
| rs1558902 | 16 | 53803574 | A/T | *FTO* | BMI | [48] |
| rs8050136 | 16 | 53816275 | A/C | *FTO* | BMI | [49] |
| rs17817449 | 16 | 53813367 | G/T | *FTO* | BMI | [56] |
| rs10938397 | 4 | 45182527 | A/G | *GNPDA2* | BMI | [48] |
| rs29941 | 19 | 34309532 | C/T | *KCTD15* | BMI | [48] |
| rs11142387 | 9 | 72998332 | A/C | *KLF9* | BMI | [45] |
| rs2890652 | 2 | 142959931 | C/T | *LRP1B* | BMI | [48] |
| rs2183825 | 9 | 28412375 | C/T | *LRRN6C* | BMI | [48] |
| rs2241423 | 15 | 68086838 | A/G | *MAP2K5* | BMI | [48] |
| rs17782313 | 18 | 57851097 | C/T | *MC4R* | BMI | [43] |
| rs2331841 | 18 | 57828637 | A/G | *MC4R* | BMI | [45] |
| rs571312 | 18 | 57839769 | G/T | *MC4R* | BMI | [48] |
| rs12970134 | 18 | 57884750 | A/G | *MC4R* | BMI | [49] |
| rs6567160 | 18 | 57829135 | C/T | *MC4R* | BMI | [56] |
| rs3817334 | 11 | 47650993 | C/T | *MTCH2* | BMI | [48] |
| rs10838738 | 11 | 47663049 | A/G | *MTCH2* | BMI | [57] |
| rs4771122 | 13 | 28020180 | A/G | *MTIF3* | BMI | [48] |
| rs2568958 | 1 | 72765116 | A/G | *NEGR* | BMI | [49] |
| rs10150332 | 14 | 79936964 | C/T | *NRXN3* | BMI | [48] |
| rs206936 | 6 | 34302869 | A/G | *NUDT3* | BMI | [48] |
| rs261967 | 5 | 95850250 | G/T | *PCSK1* | BMI | [56] |
| rs713586 | 2 | 25158008 | C/T | *POMC/ADCY3* | BMI | [48] |
| rs6545814 | 2 | 25131316 | A/G | *POMC/ADCY3* | BMI | [56] |
| rs11847697 | 14 | 30515112 | C/T | *PRKD1* | BMI | [48] |
| rs1555543 | 1 | 96944797 | A/C | *PTBP2* | BMI | [48] |
| rs2287019 | 19 | 46202172 | C/T | *QPCTL/GIPR* | BMI | [48] |
| rs4929949 | 11 | 8604593 | C/T | *RPL27A* | BMI | [48] |
| rs516636 | 1 | 177855517 | A/C | *SEC16B* | BMI | [45] |
| rs543874 | 1 | 177889480 | A/G | *SEC16B* | BMI | [48] |
| rs574367 | 1 | 177873210 | G/T | *SEC16B* | BMI | [56] |
| rs7359397 | 16 | 28885659 | C/T | *SH2B1* | BMI | [48] |
| rs13107325 | 4 | 103188709 | C/T | *SLC39A8* | BMI | [48] |
| rs987237 | 6 | 50803050 | A/G | *TFAP2B* | BMI | [48] |
| rs3810291 | 19 | 47569003 | A/G | *TMEM160* | BMI | [48] |
| rs2867125 | 2 | 622827 | A/G | *TMEM18* | BMI | [48] |
| rs7561317 | 2 | 644953 | A/G | *TMEM18* | BMI | [49] |
| rs6548238 | 2 | 634905 | C/T | *TMEM18* | BMI | [57] |
| rs1514175 | 1 | 74991644 | C/T | *TNNI3K* | BMI | [48] |
| rs4836133 | 5 | 124332103 | A/C/G | *ZNF608* | BMI | [48] |
| rs4607103 | 3 | 64711904 | C/T | *ADAMTS9* | T2D | [60] |
| rs11708067 | 3 | 123065778 | A/G | *ADCY5* | T2D | [35] |
| rs516946 | 8 | 41519248 | A/G | *ANK1* | T2D | [44] |
| rs459193 | 5 | 55806751 | C/T | *ANKRD55* | T2D | [44] |
| rs2028299 | 15 | 90374257 | A/C | *AP3S2/C15orf38* | T2D | [69] |
| rs7202877 | 16 | 75247245 | G/T | *BCAR1* | T2D | [44] |
| rs243021 | 2 | 60584819 | C/T | *BCL11A* | T2D | [53] |
| rs7172432 | 15 | 62396389 | A/G | *C2CD4A/B* | T2D | [59] |
| rs2925757 | 2 | 161101169 | C/T | *CAPN10* | T2D | [55] |
| rs73040004 | 12 | 4362909 | C/T | *CCND2* | T2D | [44] |
| rs12779790 | 10 | 12328010 | A/G | *CDC123/CAMK1D* | T2D | [60] |
| rs7756992 | 6 | 20679709 | A/G | *CDKAL1* | T2D | [34] |
| rs2206734 | 6 | 20694884 | A/G | *CDKAL1* | T2D | [45] |
| rs7754840 | 6 | 20661250 | C/G | *CDKAL1* | T2D | [46] |
| rs9356744 | 6 | 20685486 | C/T | *CDKAL1* | T2D | [56] |
| rs7513574 | 1 | 65135341 | G/T | *CDKN2A/2B* | T2D | [5] |
| rs10965250 | 9 | 22133284 | A/G | *CDKN2A/2B* | T2D | [53] |
| rs10811661 | 9 | 22134094 | C/T | *CDKN2A/2B* | T2D | [46] |
| rs2383208 | 9 | 22132076 | A/G | *CDKN2A/2B* | T2D | [53] |
| rs1552224 | 11 | 72433098 | G/T | *CENTD2* | T2D | [53] |
| rs13292136 | 9 | 81952128 | C/T | *CHCHD9* | T2D | [53] |
| rs17168486 | 7 | 14898282 | C/T | *DGKB* | T2D | [44] |
| rs2191349 | 7 | 15064309 | G/T | *DGKB/TMEM195* | T2D | [35] |
| rs5945326 | X | 152899922 | A/G | *DUSP9* | T2D | [53] |
| rs6467136 | 7 | 127164958 | A/G | *GCC1/PAX4* | T2D | [34] |
| rs8108269 | 19 | 46158513 | G/T | *GIPR* | T2D | [44] |
| rs7041847 | 9 | 4287466 | A/G | *GLIS3* | T2D | [34] |
| rs13389219 | 2 | 165528876 | C/T | *GRB14* | T2D | [44] |
| rs3923113 | 2 | 165501849 | G/T | *GRB14* | T2D | [44] |
| rs2334499 | 11 | 1696849 | C/T | *HCCA2* | T2D | [40] |
| rs7087591 | 10 | 94473629 | A/G | *HHEX* | T2D | [34] |
| rs1111875 | 10 | 94462882 | A/G | *HHEX* | T2D | [46] |
| rs5015480 | 10 | 94465559 | C/T | *HHEX* | T2D | [53] |
| rs7178572 | 15 | 77747190 | A/G | *HMG20A* | T2D | [69] |
| rs7177055 | 15 | 77832762 | A/G | *HMG20A* | T2D | [44] |
| rs1531343 | 12 | 66174894 | C/G | *HMGA2* | T2D | [53] |
| rs7957197 | 12 | 121460686 | A/T | *HNF1A* | T2D | [53] |
| rs757210 | 17 | 36096515 | A/G | *HNF1B* | T2D | [58] |
| rs4430796 | 17 | 36098040 | A/G | *HNF1B* | T2D | [46] |
| rs6017317 | 20 | 42946966 | G/T | *HNF4A* | T2D | [34] |
| rs1800961 | 20 | 43042364 | C/T | *HNF4A* | T2D | [70] |
| rs10882066 | 10 | 94224746 | A/G | *IDE* | T2D | [58] |
| rs4402960 | 3 | 185511687 | G/T | *IGF2BP2* | T2D | [46] |
| rs1470579 | 3 | 185529080 | A/C | *IGF2BP2* | T2D | [46] |
| rs2943641 | 2 | 227093745 | C/T | *IRS1* | T2D | [46] |
| rs7578326 | 2 | 227020653 | A/G | *IRS1* | T2D | [53] |
| rs849134 | 7 | 28196222 | A/G | *JAZF1* | T2D | [53] |
| rs1635852 | 7 | 28189411 | C/T | *JAZF1* | T2D | [8] |
| rs864745 | 7 | 28180556 | A/G | *JAZF1* | T2D | [60] |
| rs5215 | 11 | 17408630 | C/T | *KCNJ11* | T2D | [46] |
| rs1535500 | 6 | 39284050 | G/T | *KCNK16/KCNK17* | T2D | [34] |
| rs163184 | 11 | 2847069 | G/T | *KCNQ1* | T2D | [46] |
| rs2237897 | 11 | 2858546 | C/T | *KCNQ1* | T2D | [51] |
| rs231362 | 11 | 2691471 | C/T | *KCNQ1* | T2D | [53] |
| rs2237892 | 11 | 2839751 | C/T | *KCNQ1* | T2D | [59] |
| rs972283 | 7 | 130466854 | A/G | *KLF14* | T2D | [53] |
| rs10842994 | 12 | 27965150 | C/T | *KLHDC5* | T2D | [44] |
| rs824248 | 9 | 28772700 | A/T | *LINGO2* | T2D | [8] |
| rs6815464 | 4 | 1309901 | C/G | *MAEA* | T2D | [34] |
| rs10923931 | 1 | 120517959 | G/T | *NOTCH2* | T2D | [60] |
| rs3786897 | 19 | 33893008 | A/G | *PEPD* | T2D | [34] |
| rs1801282 | 3 | 12393125 | C/G | *PPARG* | T2D | [46] |
| rs8042680 | 15 | 91521337 | A/C | *PRC1* | T2D | [53] |
| rs340874 | 1 | 214159256 | A/G | *PROX1* | T2D | [35] |
| rs831571 | 3 | 64048297 | C/T | *PSMD6* | T2D | [34] |
| rs17584499 | 9 | 8879118 | C/T | *PTPRD* | T2D | [50] |
| rs117767867 | 17 | 6946330 | C/T | *SLC16A11* | T2D | [8] |
| rs13342232 | 17 | 6945940 | A/G | *SLC16A11* | T2D | [8] |
| rs13342692 | 17 | 6946287 | C/T | *SLC16A11* | T2D | [8] |
| rs75418188 | 17 | 6945483 | C/T | *SLC16A11* | T2D | [8] |
| rs75493593 | 17 | 6945087 | G/T | *SLC16A11* | T2D | [8] |
| rs13266634 | 8 | 118184783 | C/T | *SLC30A8* | T2D | [35] |
| rs3802177 | 8 | 118185025 | C/T | *SLC30A8* | T2D | [42] |
| rs4523957 | 17 | 2208899 | G/T | *SMG6/SRR* | T2D | [50] |
| rs1359790 | 13 | 80717156 | C/T | *SPRY2* | T2D | [39] |
| rs16861329 | 3 | 186666461 | C/T | *ST6GAL1* | T2D | [69] |
| rs7903146 | 10 | 114758349 | C/T | *TCF7L2* | T2D | [35] |
| rs12243326 | 10 | 114788815 | C/T | *TCF7L2* | T2D | [35] |
| rs4506565 | 10 | 114756041 | A/T | *TCF7L2* | T2D | [35] |
| rs7901695 | 10 | 114754088 | A/C/G/T | *TCF7L2* | T2D | [35] |
| rs10770141 | 11 | 2193840 | A/G | *TH* | T2D | [46] |
| rs11899863 | 2 | 43618819 | C/T | *THADA* | T2D | [53] |
| rs7578597 | 2 | 43732823 | C/T | *THADA* | T2D | [60] |
| rs2796441 | 9 | 84308948 | C/T | *TLE1* | T2D | [44] |
| rs1052248 | 6 | 31556581 | A/T | *TNF* | T2D | [52] |
| rs11575839 | 6 | 31557791 | C/T | *TNF* | T2D | [52] |
| rs896854 | 8 | 95960511 | A/G | *TP53INP1* | T2D | [53] |
| rs7961581 | 12 | 71663102 | C/T | *TSPAN8/LGR5* | T2D | [60] |
| rs6780569 | 3 | 23198484 | A/G | *UBE2E2* | T2D | [59] |
| rs1801214 | 4 | 6303022 | A/C/G/T | *WFS1* | T2D | [53] |
| rs4688985 | 4 | 6285715 | A/G | *WFS1* | T2D | [46] |
| rs4457053 | 5 | 76424949 | A/G | *ZBED3* | T2D | [53] |
| rs9470794 | 6 | 38106844 | C/T | *ZFAND3* | T2D | [34] |
| rs11634397 | 15 | 80432222 | A/G | *ZFAND6* | T2D | [53] |
| rs6499500 | 16 | 71348415 | C/G | *FTSJD1/CALB2* | GDM | [5] |
| rs4607517 | 7 | 44235668 | A/G | *GCK* | GDM | [35] |
| rs12898654 | 15 | 68128117 | C/G | *LBXCOR1* | GDM | [35] |
| rs1387153 | 11 | 92673828 | C/T | *MTNR1B* | GDM | [53] |
| rs10830963 | 11 | 92708710 | C/G | *MTNR1B* | GDM | [29] |
| rs10830962 | 11 | 92698427 | C/G | *MTNR1B* | GDM | [5] |
| rs187230 | 3 | 171270624 | A/C | *PLD1* | GDM | [5] |
| rs391300 | 17 | 2216258 | A/G | *SRR* | GDM | [54] |
| rs7501939 | 17 | 36101156 | C/T | *TCF2* | GDM | [4] |
| rs9395950 | 6 | 54244192 | A/G | *TINAG* | GDM | [5] |
| rs7554506 | 1 | 202944146 | A/G | *ADIPOR1* | Pregnancy trait | [52] |
| rs2041139 | 12 | 1901461 | A/G | *ADIPOR2* | Pregnancy trait | [52] |
| rs6954897 | 7 | 22750220 | A/G | *IL6* | Pregnancy trait | [52] |
| rs2227306 | 4 | 74607055 | C/T | *IL8* | Pregnancy trait | [52] |
| rs2886920 | 4 | 74626945 | C/T | *IL8* | Pregnancy trait | [52] |
| rs2167270 | 7 | 127881349 | A/G | *LEP* | Pregnancy trait | [47] |
| rs1137100 | 1 | 66036441 | A/G | *LEPR* | Pregnancy trait | [47] |
| rs1171278 | 1 | 65988146 | C/T | *LEPR* | Pregnancy trait | [52] |
| rs1627238 | 1 | 65986079 | C/T | *LEPR* | Pregnancy trait | [52] |
| rs1423096 | 19 | 7739177 | A/G | *RETN* | Pregnancy trait | [52] |
| rs1884082 | 14 | 95078677 | G/T | *SERPINA3* | Pregnancy trait | [33] |
| rs10488683 | 11 | 18053545 | A/G | *TPH1* | Pregnancy trait | [41] |
| rs2056246 | 11 | 18051446 | G/T | *TPH1* | Pregnancy trait | [41] |
| rs508924 | 11 | 18060900 | G/T | *TPH1* | Pregnancy trait | [41] |
| rs685249 | 11 | 18060587 | G/T | *TPH1* | Pregnancy trait | [42] |
| SNPs are ordered by related trait and gene. **Alleles** are reported according to NCBI data. | | | | | | |
